# Evaluation of Hydrogen Embrittlement’s Effects on the Impact Toughness of Martensitic Ultra-High-Strength Steels as a Function of the Cathodic Charging Time

**DOI:** 10.3390/ma18040764

**Published:** 2025-02-09

**Authors:** Julio C. Villalobos, Arnoldo Bedolla-Jacuinde, Álvaro Torres-Islas, Melina Velasco-Plascencia, Heriberto Villanueva, Hugo Rojas, Adrian Del-Pozo

**Affiliations:** 1 Tecnológico Nacional de México, I.T. Morelia, Avenida Tecnológico No. 1500, Col. Lomas de Santiaguito, Morelia 58120, Michoacán, Mexico; julio.vb@morelia.tecnm.mx (J.C.V.); melina.vp@morelia.tecnm.mx (M.V.-P.); 2 Instituto de Investigacion en Metalurgia y Materiales, Universidad Michoacana de San Nicolás de Hidalgo, Avenida Francisco J. Mujica S/N, Edificio U, Ciudad Universitaria, Morelia 58030, Michoacán, Mexico; arnoldo.bedolla@umich.mx (A.B.-J.); heriberto.villanuevap@docentes.uaem.edu.mx (H.V.); 3 Facultad de Ciencias Quimicas e Ingenieria, Universidad Autónoma del Estado de Morelos, Av. Universidad 1001, Col. Chamilpa, Cuernavaca 62210, Morelos, Mexico; alvaro.torres@uaem.mx; 4 Instituto de Ciencias Fisicas, Universidad Nacional Autonoma de Mexico, Cuernavaca 62210, Morelos, Mexico; hugo.rojas@icf.unam.mx

**Keywords:** hydrogen embrittlement, mechanical properties, ultra-high-strength steels, heat treatments

## Abstract

This study investigates the hydrogen embrittlement (HE) susceptibility of two martensitic ultra-high-strength steel (M-UHSS) grades, focusing on their impact toughness and microhardness behavior following different durations of hydrogen cathodic charging (1, 2, and 4 h). While some mechanisms, such as the interaction between microstructural defects and hydrogen, are well established, the effects of hydrogen on the absorbed energy during impact tests or at high strain rates have been less studied. This study correlates the microstructural characteristics, Charpy-V absorbed energy, and microhardness with fractographic analysis to assess the HE susceptibility. The results show a decrease in both microhardness and toughness after one hour of charging, with the reductions ranging from 32% to 40%. However, as the charging time increased, both properties exhibited an increase, attributed to the interaction of hydrogen and its saturation on the steel’s surface. Fractographic analysis reveals a morphological change from brittle fracture to brittle fracture with localized plastic zones, driven by the interaction of hydrogen with the trapping sites within the steel. Permeability tests are conducted to quantify the hydrogen concentration, diffusion coefficients, and trapping sites. The results indicate significant hydrogen embrittlement in both steels, driven by hydrogen diffusion and accumulation in the entrapment zones, leading to increased brittleness over time. This study provides insights into the micromechanisms influencing mechanical properties and fracture behavior under hydrogen exposure.

## 1. Introduction

With advancements in the automotive industry, there has been a growing focus on researching new advances in high-strength steels (HSSs). These steels are engineered to possess high strength and good ductility, weldability, formability, and toughness [1,2]. Ultra-high-strength steels (UHSSs), owing to their exceptional properties, find extensive applications across various industries, including pressurized vessels, pipes, automobiles, ships, offshore platforms, aircraft, trains, and rocket engine housings. The widespread utilization of these steels in industrial production is attributed to their excellent characteristics. M-UHSSs are a promising option for hydrogen containers and transportation due to their excellent strength. By studying the embrittlement mechanisms, significant progress has been made in enhancing their resistance to hydrogen embrittlement (HE) [3,4,5,6].

Beyond its significant role in the automotive sector, UHSS has emerged as an excellent alternative to traditional structural materials. Using these steels leads to a substantial reduction in the material consumption, as UHSS exhibits a similar volume-to-weight ratio as mild steel but with significantly higher mechanical resistance. Incorporating microalloying elements such as Ti and V contributes to their excellent mechanical properties [7,8]. Additionally, precipitation during heat treatments further enhances these properties by preventing the movement of dislocations [9].

Furthermore, understanding the behavior of steel when in contact with hydrogen has become crucial. Hydrogen is being explored as a clean energy source to address environmental pollution. Consequently, assessing the compatibility of steel with hydrogen is of great importance.

UHSS has remarkable mechanical properties, as reported in the literature. However, it is highly susceptible to hydrogen embrittlement (HE) [10]. The hydrogen entry mechanism and diffusion through steel depend on the microstructural characteristics and exposure time in hydrogen-rich environments. The development of hydrogen embrittlement in an aqueous medium begins with the entry of hydrogen into the metals by the electrochemical reduction of hydrogen-containing species. The mechanisms in acid solutions involve the reduction of a hydrogen ion to produce adsorbed hydrogen atoms (Hads). Then, two reactions can occur in parallel: the electrochemical reaction of electrochemical desorption (Heyrovsky reaction) to produce a hydrogen molecule and the entry of the atomic hydrogen into the metal [11,12]. The absorption of hydrogen could be caused by corrosion mechanisms, cathodic protection, or welding processes. Martensitic steels are highly susceptible to HE due to their high dislocation density, which is considered an irreversible hydrogen trap, resulting in critical effects such as cracking and a reduction in ductility [13,14]. The effect of HE has been extensively studied in various environments, such as gas phase or electrolytic solution; both processes involve adsorption, absorption, diffusion, and trapping processes [11]. Depending on the interaction between hydrogen and irreversible traps, three principal HE mechanisms have been proposed in the literature, namely hydrogen-enhanced decohesion (HEDE), hydrogen-enhanced plasticity (HELP), and adsorption-induced dislocation emission (AIDE) [11,15,16,17,18,19]. These mechanisms have been associated with costly and catastrophic failures, underscoring the importance of studying materials in contact with hydrogen to understand their behavior for subsequent implementation.

In understanding of the mechanisms of hydrogen embrittlement in UHSS, there are many factors that influence the susceptibility of brittle failures, such as microstructure, internal stress, microstructural defects, environmental conditions, and the number of hydrogen trapping sites [20]. These factors affect the mechanical properties of the steels, leading to a transition from ductile to brittle behavior once the critical hydrogen concentration is reached, as noted in previous studies [21,22,23,24]. This transition is primarily caused by mechanisms such as hydrogen-enhanced localized plasticity (HELP) and hydrogen-enhanced decohesion (HEDE). The mechanisms of hydrogen embrittlement have been widely studied, evaluating the macroscopic mechanical properties, such as the tensile test, fatigue, and microhardness, and microscopic mechanical properties, such as the microhardness and nanoindentation with and without hydrogen cathodic charging [24,25,26]. From these tests, several theories have been proposed; however, a unified theory has not yet been fully established [22,27]. As an example, the variation in the embrittlement index [28,29,30] should be calculated by the area reduction of the specimens after the tensile test and hydrogen charge. However, it is necessary to understand the hydrogen effect on all the aforementioned mechanical properties and measurables [28,29,30].

An important mechanical property that should be considered is the impact toughness and its variation caused by hydrogen’s effect on UHSS. The Charpy-V tests have been less studied after hydrogen charging. However, the hydrogen–dislocation interaction and, therefore, the hydrogen embrittlement micromechanisms are unclear due to the high strain induced in the sample by the impact during tests. The hydrogen–dislocation synergistic effect should be analyzed under high-strain conditions to establish the principal embrittlement mechanisms involved in the fracture and absorbed energy in UHSS. In the literature, there are some research works that reported brittle fractures after hydrogen charging and impact tests [31,32,33,34]. Otherwise, some other research reported cracking after permeability tests [35], indicating that hydrogen charging as a function of time could cause internal effects such as cracking and cavities that induce the bulk material to be compromised before the impact tests. The present research aims to develop two UHSS alloys and assess their resistance to HE at three different hydrogen charging times and its effect on the fracture behavior of impact test samples.

## 2. Materials and Methods

### 2.1. Material

Two ultra-high-strength steels (UHSSs), designated as L1 and L2, were conceived and produced using a high-vacuum induction furnace. The chemical composition of both steels was determined using spark spectroscopy, and the results are presented in Table 1. It is noteworthy that Mo, V, Ti, and Nb play significant roles as carbo-nitride formers, contributing to the enhanced mechanical strength through mechanisms such as solid solution and inhibition of austenitic grain growth during thermomechanical treatment.

Conversely, elements like Al have a tendency to form transitional carbides during tempering treatments, potentially inducing secondary hardening. These phenomena are crucial to assess their effects on the hydrogen embrittlement susceptibility.

**Figure 1:**
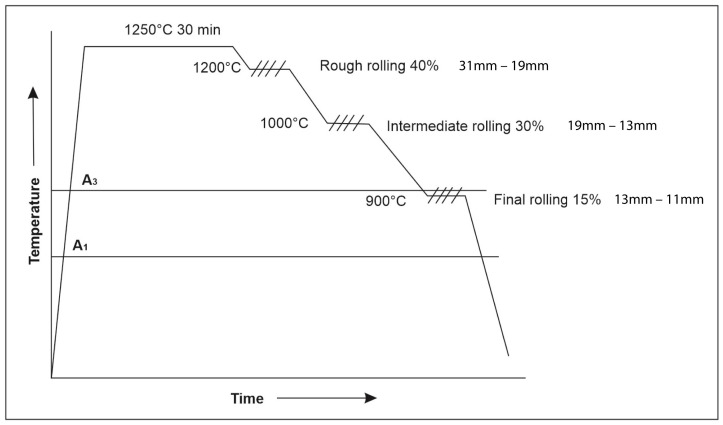
Diagram of the reduction percentages and temperatures for the thermomechanical processing of the UHSSs.

After obtaining the UHSS alloys, the steels were reheated to a temperature of 1200 °C for 4 h and then subjected to thermomechanical treatment in multiple passes (refer to Figure 1) to enhance their mechanical properties. A thickness reduction of 23 mm was performed in several stages, to reach the final thickness of 11 mm, as follows: rough rolling at 1150 °C above the non-recrystallization temperature (T_nr_), with a reduction percentage of approximately 41.6%; intermediate rolling at 1000 °C below the Tnr, with a reduction percentage of about 33.3%; and finally, the last rolling at a temperature slightly above the austenitic transformation temperature (A_c3_), at 900 °C, with a reduction percentage of approximately 25%. Following the thermomechanical process, the steels were air-cooled with a cooling rate of 15 °C/s, and thereafter, the steels (L1 and L2) were considered to be in their as-received condition. Subsequently, specimens of the treated steels were machined to the required dimensions for the impact Charpy-V tests and microhardness measurements.

### 2.2. Metallography

Both as-received steels (L1 and L2) underwent metallographic preparation to determine their microstructure. The procedure involved cutting the L1 and L2 samples into approximately 10 mm × 10 mm pieces, which were then polished to achieve a mirror finish. This polishing process utilized SiC sandpaper with different granulometries, and a final mirror finish was achieved using alumina (Al_2_O_3_) with particle sizes of 3 µm and 1 µm.

The microstructure was revealed by immersing the prepared samples in a 5% Nital reagent solution for 15 s. Following the revelation of the microstructure, the samples underwent metallographic characterization using scanning electron microscopy (SEM).

### 2.3. Microhardness and Impact Tests

The preparation of the specimens for the Vickers microhardness tests involved cutting samples from both steels to a size of 10 mm × 10 mm, followed by polishing with different sandpapers until reaching 1000-grit sandpaper. In accordance with the ASTM E384 Standard [36], twenty-five indentations were made at a load of 1000 g for 15 s under each study condition.

**Figure 2:**
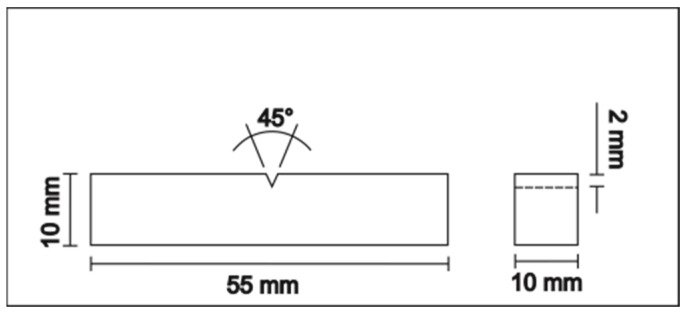
Scheme of the impact test specimen.

For the impact Charpy-V tests on the UHSSs (L1 and L2), specimens were manufactured following the ASTM E23 Standard [37] to determine the impact toughness. Three impact tests were conducted for each study condition on the steel, with Figure 2 illustrating the dimensions used for the impact specimens. Both the Vickers microhardness and impact Charpy-V tests were performed in the as-received condition (without hydrogen charging) and after hydrogen charging for 1, 2, and 4 h. This approach allowed the evaluation of the impact of the hydrogen embrittlement susceptibility on the mechanical properties.

### 2.4. Hydrogen Cathodic Charging

The hydrogen cathodic charging of samples L1 and L2 was conducted in a solution consisting of 0.5 M H_2_SO_4_ + 0.2 g As_2_O_3_, with a current density of 140 mA/cm^2^. The hydrogen charging was performed at room temperature using a Tekpower TP3005D power supply (Tekpower, San Diego, CA, USA) with an error range of 1.5 E + 2 W, as well as a platinum LD-Teemm electrode serving as the cathode. Three different charging times were employed: 1 h, 2 h, and 4 h, respectively. The objective was to facilitate hydrogen diffusion in the crystal lattice and assess the susceptibility to hydrogen embrittlement under each condition.

**Figure 3:**
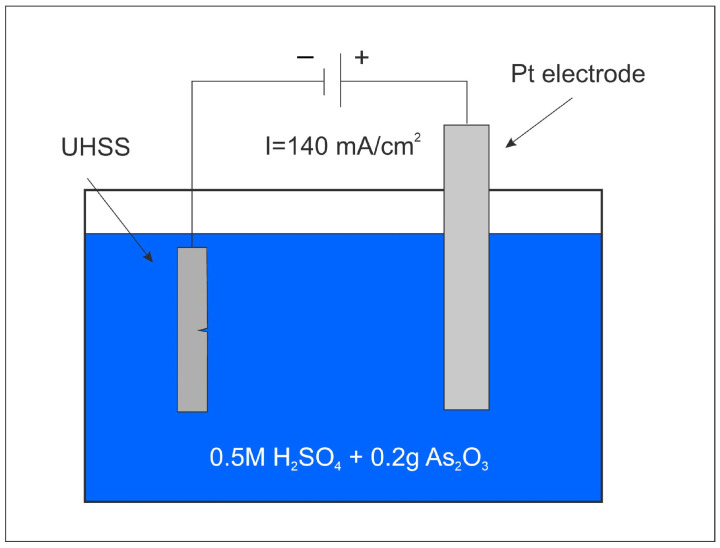
Diagram of the experimental setup for cathodic hydrogen charging.

The tests were carried out in a cathodic charging cell (refer to Figure 3). The impact Charpy-V test sample and the microhardness sample were submerged in the solution to enhance the hydrogen concentration at the UHSS surface and facilitate diffusion into the samples. Subsequent to the hydrogen charging, the samples were immediately tested, and the correlation between the changes in their mechanical properties and the hydrogen charging time was analyzed.

### 2.5. Hydrogen Permeability Test

The hydrogen permeability tests were conducted in accordance with the ASTM G-148 Standard [38]. The procedure involved employing a charging cell containing a 0.5 M H_2_SO_4_ + 0.2g As_2_O_3_ solution, with a current density of 140 mA/cm^2^ applied. Simultaneously, an oxidation cell filled with 0.1 N NaOH was utilized, applying an anodic potential of 300 mV with respect to a calomel electrode. Membranes with a thickness of 1 mm, prepared from L1 and L2 steels with a surface condition of 600 SiC grit, were used in the tests.

The current density on the anodic cell was monitored over time to obtain the permeability curves. Following the ASTM G-148 Standard, hydrogen permeability parameters such as the hydrogen apparent concentration (C_app_), effective diffusion coefficient (D_eff_), and hydrogen flux (J_ss_L) were determined.

## 3. Results

### 3.1. Microstructural Characterization

**Figure 4:**
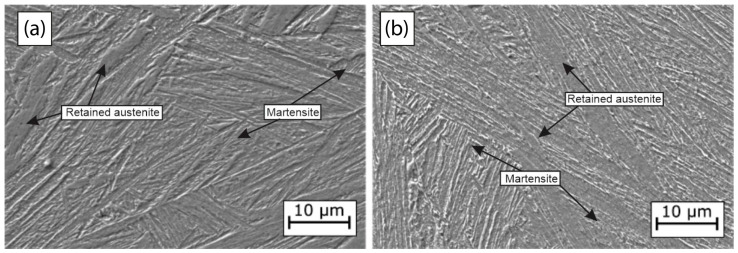
Micrographs of the two UHSSs with different Mo and V contents: (**a**) L1 and (**b**) L2.

In Figure 4, micrographs obtained from the scanning electron microscope are presented. Figure 4a displays the micrograph obtained from the L1 steel, while Figure 4b depicts the micrograph of the L2 steel. Both steels exhibit a predominantly martensitic morphology, attributed mainly to the high Mo content and the deformation process [39]. In both cases, lath martensite, along with small amounts of retained austenite, is observed. The grains appear randomly oriented at various angles, resulting in a wide range of grain boundaries. Analyzing the steel L2 (Figure 4b) reveals martensite laths with smaller widths compared to the steel L1 (Figure 4a). The misorientation of the grain angles is linked to the distribution of dislocations, providing sites for hydrogen segregation and contributing to the number of entrapment sites [40].

**Figure 5:**
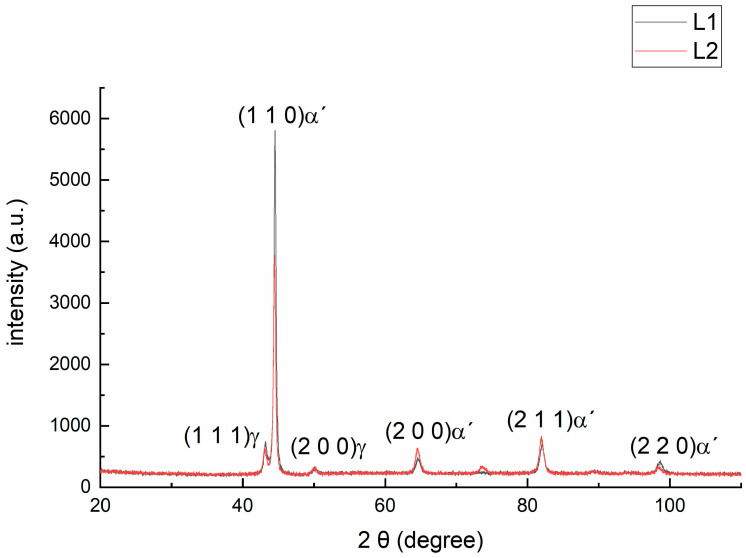
X-ray diffractogram of the L1 steel and L2 steel scans using Cu K_α_ radiation.

A typical XRD profile of steels L1 and L2 is shown in Figure 5, obtained at room temperature using Cu Kα radiation. The individual diffraction planes corresponding to α’-martensite and γ-retained austenite are clearly visible and marked in the figure. Peaks corresponding to α’-martensite are observed in the (1 1 0) (2 0 0) (2 1 1) and (2 2 0) planes, indicating the presence of α’-martensite. Additionally, peaks corresponding to the retained austenite (RA) phase are observed in the planes (1 1 1) and (2 0 0), formed during the deformation process caused by hot rolling. Both diffractograms display the same characteristic peaks, differing only in the intensity (a.u.), which can be attributed to the similarity in the microstructure of both steels. These observations align with the measurements conducted in XRD by Rodríguez et al. during the characterization of α’-martensite and retained austenite (RA) in martensitic steels AISI 5160, 9260, and D2 [41,42].

### 3.2. Microhardness Tests

In Table 2, the results of the microhardness tests without hydrogen charging and with hydrogen charging are presented. The L1 steel in the as-received condition showed a higher microhardness value of about 584 HV, characteristic of a martensite microstructure; and after 1 h and 4 h of hydrogen charging time, it was observed to have a reduction of around 397 HV, which represents a 33% hydrogen embrittlement index [43]. On the other hand, the value obtained after 2 h of hydrogen charging was 465 HV; this reduction represents a hydrogen embrittlement index of about 20%.

Conversely, the steel L2, in the as-received condition, showed a value of about 577 as similar behavior was described in the L1 steel. At 1 h of hydrogen charging time, the value obtained was 347 HV, which represents a 39% hydrogen embrittlement index. However, with the increase in the hydrogen charging time, the values of the microhardness showed a slight increase from 374 HV up to 481 HV, which represents a hydrogen embrittlement index of about 35% and 17%, respectively, for 2 h and 4 h.

**Figure 6:**
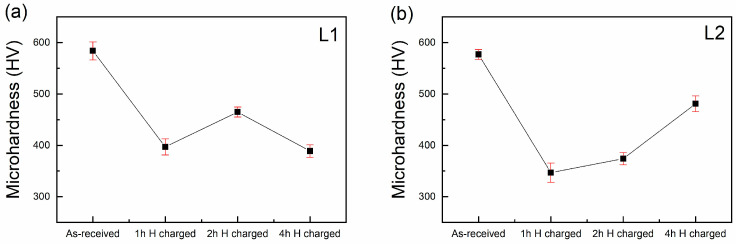
Microhardness test results of the (**a**) L1 and (**b**) L2 steels with and without hydrogen charging.

In general terms, the L1 and L2 steels showed a higher embrittlement index after 1 h of hydrogen charging, attributed to the interaction of hydrogen with the metallic matrix, causing localized stress on the surface of the samples [44]. However, sample L1 showed a slight increase in its values of microhardness at 2 h of hydrogen charging but at 4 h tended to decrease. This fluctuation in the microhardness values could attributed to the hydrogen trapped around dislocations that can retard the dislocations’ motion. Otherwise, it reports an increase in hardness for an interaction of dislocation–dislocation close to the indents where hydrogen promoted dislocation nucleation [45,46,47]. On the other hand, sample L2 showed an increase in the microhardness values as the hydrogen charging time increases, caused by localized plasticity, which induces localized hardening by deformation [48]. Figure 6 illustrates the microhardness test results for both steels, showcasing a general decrease in microhardness.

### 3.3. Charpy-V Impact Tests

The hydrogen charging process results in the trapping of hydrogen within the matrix, generating internal stresses that heighten the susceptibility to cracking even in the absence of external mechanical stress, consistent with observations by M. Dávila-Pérez et al. [35]. The assessment of the high-stress rates through Charpy-V impact tests can provide valuable insights for understanding the HE phenomenon in these steels [49].

**Figure 7:**
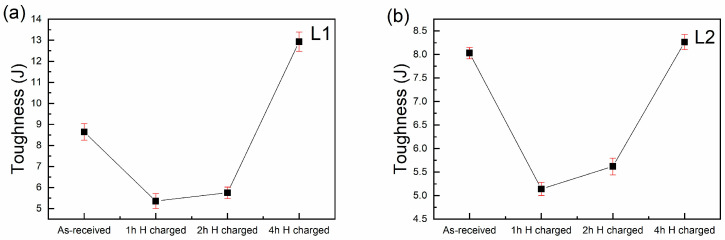
Impact test toughness plot of the (**a**) L1 and (**b**) L2 steels with and without hydrogen charging.

The results of the impact tests on the two steels, both with and without hydrogen charging, are illustrated in Figure 7, depicting data collected at 1 h, 2 h, and 4 h intervals. The Charpy-V impact tests were employed to assess the ductile–brittle behavior of the steel concerning the changes induced in the matrix due to hydrogen’s effect when interacting with the entrapment sites. These tests are recognized for their ability to evaluate the absorbed energy, which is closely associated with ductile–brittle behavior [50,51,52,53]. The L1 and L2 steels showed similar behavior in terms of their impact toughness values. At 1 h of hydrogen charging, the reduction in absorbed energy is evident, representing an embrittlement of about 35%; as the hydrogen charging time increases, the absorbed energy increased at 2 h and 4 h, with this last condition showing a higher impact toughness that is coherent with the results observed and discussed in the fractography section. It is important to note that a greater effect of hydrogen on the mechanical properties was observed at 1 h of hydrogen charging, as is discussed in further sections of this manuscript, while the dominance of the HEDE mechanism is predominant at this condition of hydrogen charging. On the other hand, the predominant mechanism changes with an increase in the hydrogen charging time toward the HELP mechanism.

**Figure 8:**
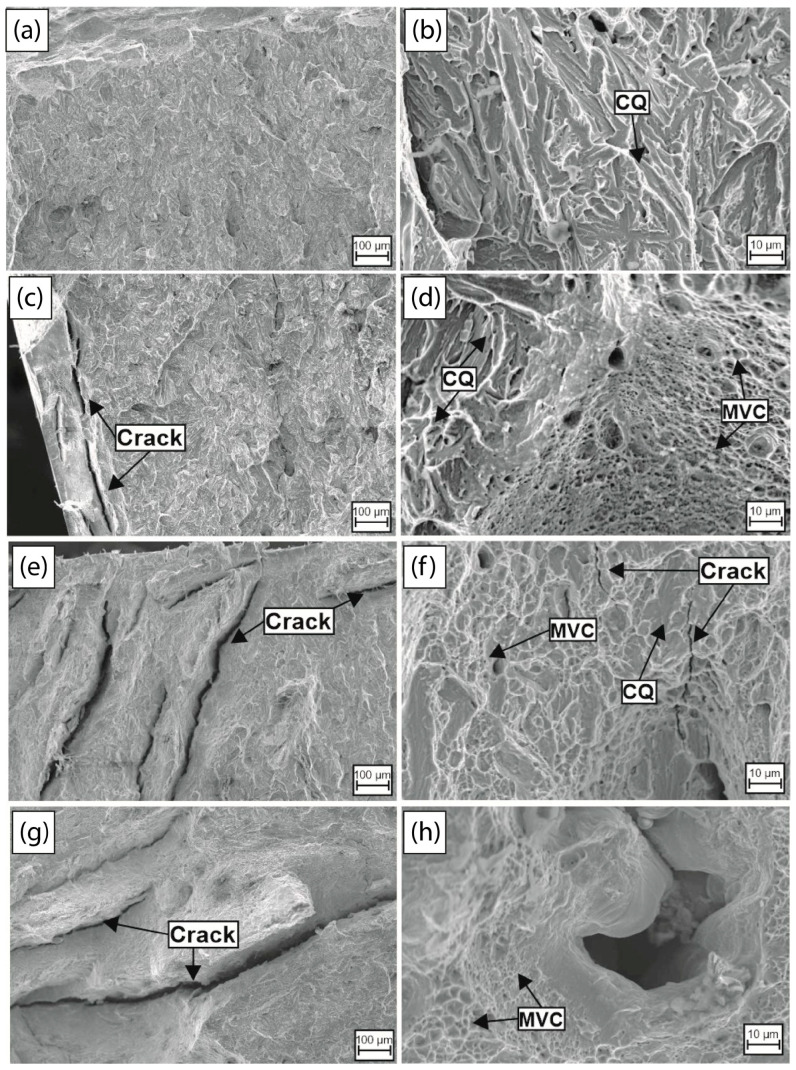
Impact fractographs of the L1 steel in the (**a**,**b**) as-received condition, and after (**c**,**d**) 1 h, (**e**,**f**) 2 h and (**g**,**h**) 4 h of hydrogen cathodic charging.

It is crucial to analyze the fracture surface of the L1 and L2 samples tested in order to understand the mechanisms of HE and its evolution as a function of the hydrogen charging time. Figure 8 presents the fractographs in the peripheral zone of the steel L1 under as-received conditions, and after 1 h, 2 h, and 4 h of hydrogen charging time, at lower and higher magnification. In the as-received condition, an essentially brittle behavior of the fracture surface is observed at lower magnifications (Figure 8a), without the presence of cracks or voids on the surface of the sample. On the other hand, at higher magnification (Figure 8b), cleavage micromechanisms of fracture are observed, with characteristic patterns such as river marks, faceted surfaces and transgranular fractures, which are evident. These characteristics are indicative of the low absorbed energy capability of the L1 steel and this behavior is coherent with the values obtained in the Charpy-V test under this condition (8.5 J of absorbed energy), which causes faster cracking without the presence of plastic deformation.

Upon analyzing the surface fracture of the sample tested after 1 h of hydrogen charging time, a brittle surface appearance with the presence of cracks on the left edge of the sample is observed at lower magnification (Figure 8c), with sizes about 200 µm approximately, caused by the diffusion of hydrogen carried out near the surface. This is because the time is not enough for hydrogen to diffuse up to the center of the sample [54]. At higher magnification (Figure 8d), the fracture micromechanism observed under this hydrogen charging condition shows the presence of cleavage (CQ) and transgranular fracture characteristics of a brittle behavior [55]. However, microvoids of different sizes of about 10 µm, which are characteristic of ductile fracture caused by the presence of plastic deformation during fracture processes [56], are also observed. Under this condition, the embrittlement effect of hydrogen on the impact toughness and fracture evolution is evident because the absorbed energy was about 5.5 J, the lowest reduction in the impact toughness, and the presence of QC zones surrounded by microvoid coalescence zones (MVC) is characteristic of the HE mechanisms denominated by hydrogen-enhanced localized plasticity (HELP), which is caused by the interaction of hydrogen–dislocation during the deformation process, as reported in the literature [57,58,59].

As the hydrogen charging time increases to 2 h and 4 h, the presence of long cracks (indicated) of different sizes (approximately about 1 mm) at low magnifications (Figure 8e,g) becomes more evident on the edges and in the center of the tested samples, indicating a longer distance of hydrogen diffusion and trapping on critical sites, increasing the localized hydrogen concentration, and as a consequence, a high stress intensity caused by the saturation of the sample for a higher duration of hydrogen exposure [60]. At higher magnification (Figure 8f and Figure 8h, respectively), the presence of cleavage and transgranular fractures is considerably reduced, replaced by an increase in the presence of MVC caused by the HELP mechanisms aforementioned and voids of about 40 µm caused by the accumulation of a high hydrogen concentration [61]. It is evident that the HE susceptibility increases with a higher hydrogen charging time due to the presence of cracks in the fracture appearance. However, the absorbed energy decreases at 1 h and subsequently increases at 2 and 4 h (6 J and 13 J, respectively), corresponding to the localized plasticity mechanism caused by the enhancement of the dislocation movement by hydrogen during the deformation process [59].

**Figure 9:**
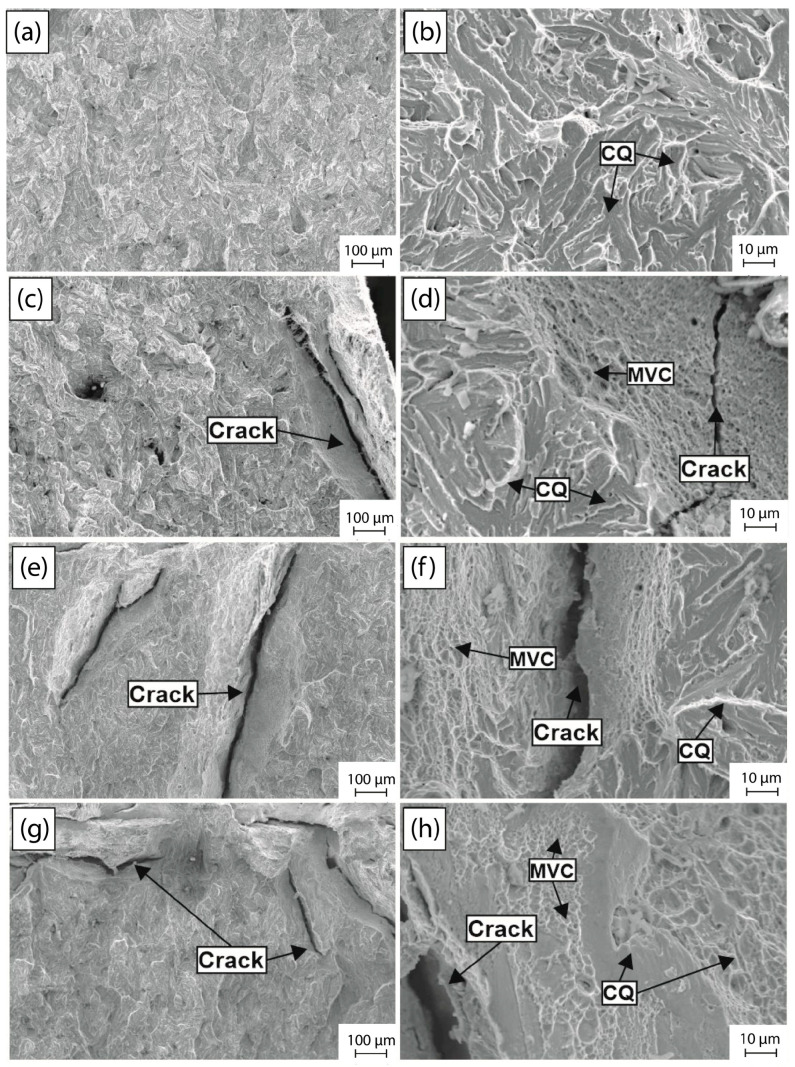
Impact fractographs of the L2 steel in the (**a**,**b**) as-received condition, and after (**c**,**d**) 1 h, (**e**,**f**) 2 h and (**g**,**h**) 4 h of hydrogen cathodic charging.

In Figure 9, fractographs corresponding to the L2 sample tested in Charpy-V are depicted. The periphery zone of the fractured surface was analyzed, in the as-received condition and after 1 h, 2 h, and 4 h of hydrogen charging time. The behavior of this L2 steel is quite similar to that observed in the L1 steel, exhibiting a brittle fracture appearance at lower magnifications in the as-received condition (Figure 9a), with micromechanisms related to cleavage (CQ), faceted surfaces and river marks (Figure 9b). However, as the hydrogen charging time increases, the presence of long cracks of approximately 1 µm is presented at lower magnifications, which indicates higher HE caused by a hydrogen-enhanced decohesion (HEDE) mechanism (Figure 9c,e,g). Analyzing the micromechanism at higher magnifications (Figure 9d,f,h), it is observed that cleavage zones (QC) are reduced; instead, microvoid mechanisms (MVC) surrounded by cracking of different sizes become more prominent. The combination of these micromechanisms is related to the enhancement of the HELP mechanisms that increase as a function of the hydrogen charging time [62]. It is important to note that a reduction in absorbed energy is observed at 1 h of hydrogen charging time. However, as the time increases, there is an increase in the absorbed energy, corroborating the localized plasticity observed in the fractographs. In general terms, the L1 showed a higher absorbed energy under all conditions compared to the L2.

### 3.4. Hydrogen Permeability

**Figure 10:**
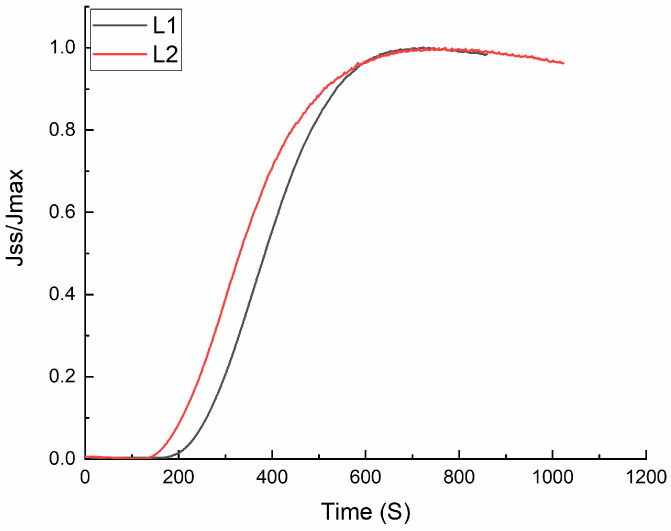
Hydrogen permeability curves obtained from the L1 and L2 steels using a double electrochemical cell method (Devanathan–Stachurski cell).

In this section, the permeability curves for the L1 and L2 steels are analyzed in order to obtain diffusion parameters such as the hydrogen flux (J_ss_L), effective diffusion (D_eff_), and hydrogen apparent concentration (C_app_). Figure 10 shows the permeability curves for these steels, and their permeability parameters are shown in Table 3. The L1 steel showed slightly higher C_app_ and lower D_eff_ hydrogen parameters, with values of about 0.48 ppm and 3.86 × 10^−6^ cm^2^/s, respectively, compared to the L2 steels; however, the difference is not significant due to the similitude of the morphology and microstructure observed, which are mainly composed by martensite and retained austenite. However, this slight difference in parameters is associated with the difference in the Mo and V contents, which promote the precipitation of carbonitrides that are strong hydrogen traps [63]. Otherwise, it is enough to observe a different behavior on the impact of the Charpy-V tests and its respective fracture morphology. The absorbed energy on the tested samples after 1 and 2 h of hydrogen charging had practically the same behavior; however, at a higher hydrogen charging time (4 h), the L2 sample presented a lower absorbed energy, which is associated with the lower hydrogen flux (J_ss_L) and higher site traps, as the permeability parameters suggest [64]. It is reported that a slight difference in the hydrogen concentration could cause a higher hydrogen embrittlement index [65]. As observed in the fractography evidence, at lower hydrogen charging times (1 and 2 h), it is predominantly a surface without the presence of cracking or voids caused by the hydrogen accumulation; at higher hydrogen charging times, the presence of cracks with longer sizes is observed, and it is more evident in the L2 steel.

In order to evaluate the hydrogen diffusion distance, the following equation was used: $x={\left(D_{eff}\cdot t\right)}^{\frac{1}{2}}$, where x represents the hydrogen diffusion distance (cm), $D_{eff}$ represents the effective coefficient diffusion (cm^2^/s) and t (s) represents the hydrogen charging time [54]. It can be observed in Table 2 that the maximum distance of hydrogen diffusion was 0.25 cm for the L2 steel, which indicates that it presented a higher hydrogen concentration at the edges of the samples, as was observed in the fractographic analysis; otherwise, as was observed, the nucleation and crack growth developed from the edges to the center of the sample. The variation in the microhardness values was due to the localized hydrogen concentration on the surface of the steels.

## 4. Discussion

Two different UHSSs were analyzed, with variations in their composition, along with variations in the cathodic hydrogen charging times. One of the main challenges in understanding the mechanisms of hydrogen embrittlement (HE) is comprehending the increased localized plasticity due to the interaction of hydrogen with dislocations, known as the HELP mechanism [66]. This interaction results in an increase in the mobility and activity of the dislocations. The growth of localized zones of plasticity, marked by the formation of MVC around cracks due to the interaction of hydrogen with dislocations, was investigated by Birnbaum [67]. The study determined that the presence of hydrogen in a solid solution reduces the mobility barriers of dislocations. The HELP mechanism is based on the dislocation mobility and slip at the crack tip [68], leading to localized material softening, as observed in the hydrogen-charged fractographs.

The energy absorbed during the impact tests (Figure 7) is influenced by the type of material fracture. Hydrogen-free steel exhibits brittle fracture characteristics, as indicated by the cleavage morphology observed in Figure 8a,b and Figure 9a,b and its low energy absorption. After one hour of cathodic charging, the absorbed energy decreases, reflecting a transition to a more brittle fracture. Surface cracks are evident due to hydrogen diffusion, and fractographic analysis reveals the formation of microvoids near the sample’s center. This suggests the synergistic interaction of hydrogen embrittlement mechanisms, specifically hydrogen-enhanced localized plasticity (HELP) and hydrogen-enhanced decohesion (HEDE).

Cracks near the surface result from material decohesion upon reaching a critical hydrogen concentration (HEDE). As hydrogen diffuses from the surface toward the material’s center, its concentration is higher near the surface, increasing the likelihood of hydrogen-induced cracking. Toward the sample’s center, the hydrogen concentration decreases, leading to a shift in the mechanisms. The microvoid morphology observed in these regions is attributed to the enhanced dislocation mobility and slip at the crack tip caused by hydrogen (HELP).

The interaction between these mechanisms reduces the energy absorbed in both steels after one hour of cathodic charging, with HEDE being the dominant mechanism under this condition (HEDE > HELP) [69,70,71]. As the charging time increases to 2 and 4 h, the absorbed energy consistently rises. This increase corresponds to a greater presence of microvoids near the sample’s center, enhancing the hydrogen-induced plasticity zones (HELP). These regions have not yet reached critical hydrogen concentration levels. The highest energy absorption is observed after 4 h of charging in the steel L1.

In both steels (L1 and L2), the dominance of the HELP mechanism grows with an increased charging time as hydrogen permeates further toward the center without reaching critical concentrations in these regions. However, the initiation of cracks confirms the rising hydrogen concentration throughout the material.

As seen in the fractographs (Figure 8 and Figure 9), there is localized softening around the crack formations due to the HELP mechanism, which has a more significant influence compared to the HEDE mechanism. The HEDE embrittlement mechanism involves a decrease in the cohesive force because hydrogen can anchor in interstitial sites, causing dilatation of the atomic lattice [67,72]. For the hydrogen-enhanced decohesion (HEDE) mechanism to occur, a sufficiently high critical hydrogen concentration must be locally reached in the metal. This process is based on the hypothesis that interstitial hydrogen reduces the material’s cohesive strength by expanding the atomic lattice [69]. The dominance of an embrittlement mechanism and extension on the material surface results from nano- and micro-level processes in the fracture zone ahead of the crack tip. The formation of MVC surrounding the cracks, intertwined with quasi-cleavage (QC) zones, is primarily driven by the dominance of the HELP mechanism. Consequently, the prominent fracture characteristics, MVC and QC, arise from the synergistic interplay of both mechanisms (HELP and HEDE), as was previously studied by M.B. Djukic et al. [33,70].

The behavior between both steels (L1 and L2) did not exhibit significant changes in susceptibility to HE, as the difference in the chemical compositions did not lead to a significant difference in the microstructural morphology. However, an increase in the severity of the HE susceptibility with longer hydrogen charging times (1, 2, and 4 h) was observed. The interaction of both mechanisms (HELP and HEDE) is associated with hydrogen trapping sites, such as carbides, non-metallic inclusions, grain boundaries, dislocations, and carbonitrides (C, N) (Ti, Nb, V), which are strong hydrogen traps, and as suggested by the permeability parameters, a higher hydrogen concentration and higher diffusion coefficient were observed to be caused by faster hydrogen diffusion and high interaction with these defects [73,74]. Longer hydrogen charging times generate higher hydrogen concentrations that reach critical levels in these areas, triggering crack initiation and subsequent fracture [75,76,77].

Analyzing surface cracks presents significant challenges due to the limited availability of direct experimental evidence. Although the mechanisms of HE, such as HEDE and HELP, have been extensively studied and linked to surface crack formation, the evidence supporting these associations is often indirect or lacks statistical validation.

A major obstacle lies in the difficulty of directly observing hydrogen atoms, tracking their movement and trapping them, and understanding their interactions with microstructural defects. Furthermore, the complex, dynamic, and multidimensional nature of HE demands experimental and computational approaches that can address varying spatial and temporal scales, which remains a persistent challenge. These limitations emphasize the importance of further research to advance the understanding of this intricate phenomenon.

## 5. Conclusions

In this study, impact tests, microhardness measurements, and permeability analyses were conducted to evaluate the behavior of hydrogen embrittlement in two UHSSs subjected to three different charging times. Both steels exhibited similar behavior, with the most significant decrease in microhardness observed in the L2 steel after 1 h of cathodic charging, showing a reduction of 39%. This reduction was attributed to the interaction of hydrogen with trapping sites. The behavior of the steels in the impact tests showed an initial decrease in absorbed energy, attributed to the dominance of the HEDE mechanism. As the charging time increased, the dominant mechanism shifted to HELP, as hydrogen permeated further into the interior without reaching critical concentrations in these regions. A slightly higher C_app_ parameter and a slightly lower D_eff_ parameter were observed for the L1 steel, with values of approximately 0.48 ppm and 3.86 × 10^−6^ cm^2^/s, respectively. Similarly, it was found that after 4 h of charging, the hydrogen diffusion distance was 0.236 cm for the L1 steel and 0.251 cm for the L2 steel. Based on these findings, it was concluded that for both steels, as the charging times increased, critical levels of hydrogen were reached, triggering crack initiation and subsequent fracture.

## Figures and Tables

**Table 1:** Chemical composition of ultra-high-strength steels (wt%).

Reference	C	Mn	Si	Ni	Cr	Al	Ti	Nb	Mo	V	Fe
L1	0.4	0.8	0.72	0.0071	0.96	0.03	0.0043	0.02	2.00	0.086	bal
L2	0.4	0.7	0.74	0.0064	0.86	0.03	0.0037	0.02	2.44	0.101	bal

**Table 2:** Microhardness test values with and without hydrogen charging.

Reference	L1 As-Received	L2 As-Received	L1 1h H Charged	L2 1h H Charged	L1 2h H Charged	L2 2h H Charged	L1 4h H Charged	L2 4h H Charged
HV prom	584	577	397	347	465	374	389	481

**Table 3:** Hydrogen permeability parameters obtained from the permeability curves.

Condition	J_ss_L (mol/cm·s) × 10^−11^	D_eff_ (cm^2^/s) × 10^−6^	C_app_ (ppm)	d_4h_ (cm)
L1	1.47	3.86	0.48	0.236
L2	1.28	4.38	0.37	0.251

## Data Availability

The original contributions presented in the study are included in the article, further inquiries can be directed to the corresponding author.
